# An optimized filling method for capillary DLS

**DOI:** 10.1016/j.mex.2019.03.006

**Published:** 2019-03-21

**Authors:** Valentina Ruseva, Hanna Jankevics, Jason Corbett

**Affiliations:** Malvern Panalytical Ltd., United Kingdom

**Keywords:** Optimized filling strategy for capillary DLS, Capillary, Dynamic light scattering, Method optimization

## Abstract

Capillary dynamic light scattering (DLS) is a new, simple and enabling technique, that increases the size range of DLS by over an order of magnitude in a cheap, disposable, but high optical quality, glass capillary. Sample loading for other capillary-based modalities, such as blood analysis, is typically achieved by dipping the capillary into the bulk sample, however, DLS is exquisitely sensitive to static scattering such as from a fluid meniscus or sample dried on the outside of the capillary and is sometimes used for extended measurement times where evaporation must be avoided. In this work, we carefully validate capillary dipping and sealing with a clay plug for DLS against reference measurements in a high quality 1 cm cuvette and then introduce a simple capillary loading scheme that reproducibly places a >3 μl sample in the correct location for a DLS measurement. We demonstrate the statistically identical characterisation of the new scheme and dipping against the reference measurements, but in sample volumes reduced by 1 and 3 orders of magnitude, respectively, key for high value applications such as pharmaceutical development where sample costs of $100 k per mg are common and in the environmental & medical sciences where samples may be difficult or unethical to collect in bulk.

• Use of the capillary method to characterize high value samples in the lowest, reproducible volume.
• Pitfalls and subsequent development of the best reproducible method.

## Graphical abstract

**Figure:**
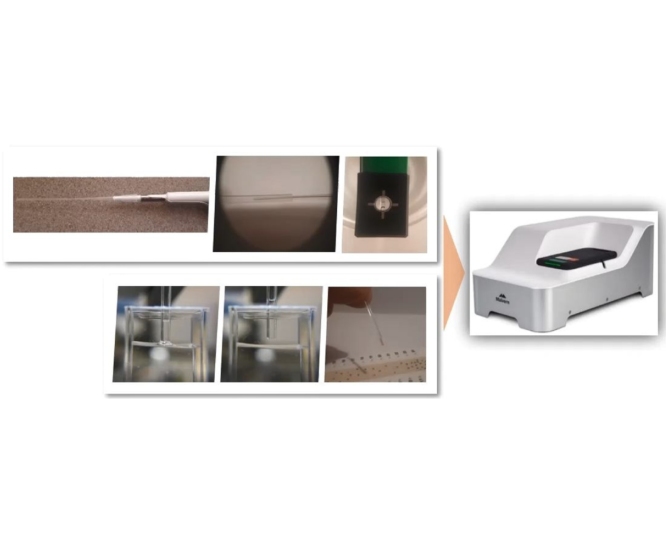

Specifications Table

**Table:** 

**Subject Area:**	*Materials Science*
**More specific subject area:**	*Dynamic light scattering*
**Method name:**	Optimized filling strategy for capillary DLS
**Name and reference of original method**	‘Capillary dynamic light scattering: continuous hydrodynamic particle size from the nano to the micro-scale’, V Ruseva, M Lyons, J Powell, J Austin, A Malm, J Corbett, Submitted COLSUA (2018) [1]
**Resource availability:**	*GELoading tips, Zetasizer Ultra, Capillary Cell*

## Method details

Dynamic light scattering [2] characterizes the relaxation time(s) associated with spatially and temporally coherent light scattered from a diffusing ensemble of particles in dispersion. The scattered light is collected at a particular detection angle and the rate at which the particles are diffusing, the diffusion co-efficient *D*, m^2^ s^−1^, is derived from the autocorrelation function of the collected photon train. The particle size is then calculated from *D*, using a Stokes equivalent sphere. The rate at which the particles diffuse is exquisitely sensitive to forms of particle motion other than diffusion, such as gravitational settling and bulk thermodynamic fluid flow in the thermally controlled cuvette. A careful analysis of the different relaxation times in the time-correlated data [1], failed to reproduce the timescales typically associated with gravitational settling, the uncertainty long assumed to be the limiting factor for the upper size limit of DLS measurements. Convective motion within the capillary cell was carefully modelled using ANSYS and the timescales associated with convective motion were identified in the correlogram. Capillary DLS seeks to suppress the convective motion within the sample, whilst maintaining excellent thermal control of the sample by conduction, with good thermal contact between a metal capillary holder and capillary outer surface, over the length of the capillary. It was discovered that 1.00 mm × 1.00 mm was an optimum size for the square capillary internal cross section [1] as a balance between the need to suppress the convective motion and reduce the sample volume, with the need to be able to reproducibly mount and align the capillary with the optical path within the instrument. The capillaries used in this work are 50 mm in length making them easy to handle, however, the 1.0 mm × 1.0 mm aperture means that care is needed in loading the sample into the capillary bore.

Dipping and sealing with a clay plug is a traditional method of capillary loading found in other measurement modalities [[3], [4], [5]]. In this work, we first validate this method for DLS where higher sample volumes and the avoidance of sample evaporation over extended measurement times is sometimes of importance. We then demonstrate the method of micro-pipetting tiny sample volumes directly into the capillary, using long reach pipette tips, for high value samples.

## Method development

### Immersion filling: ‘dipping’

**Fig. 1:**
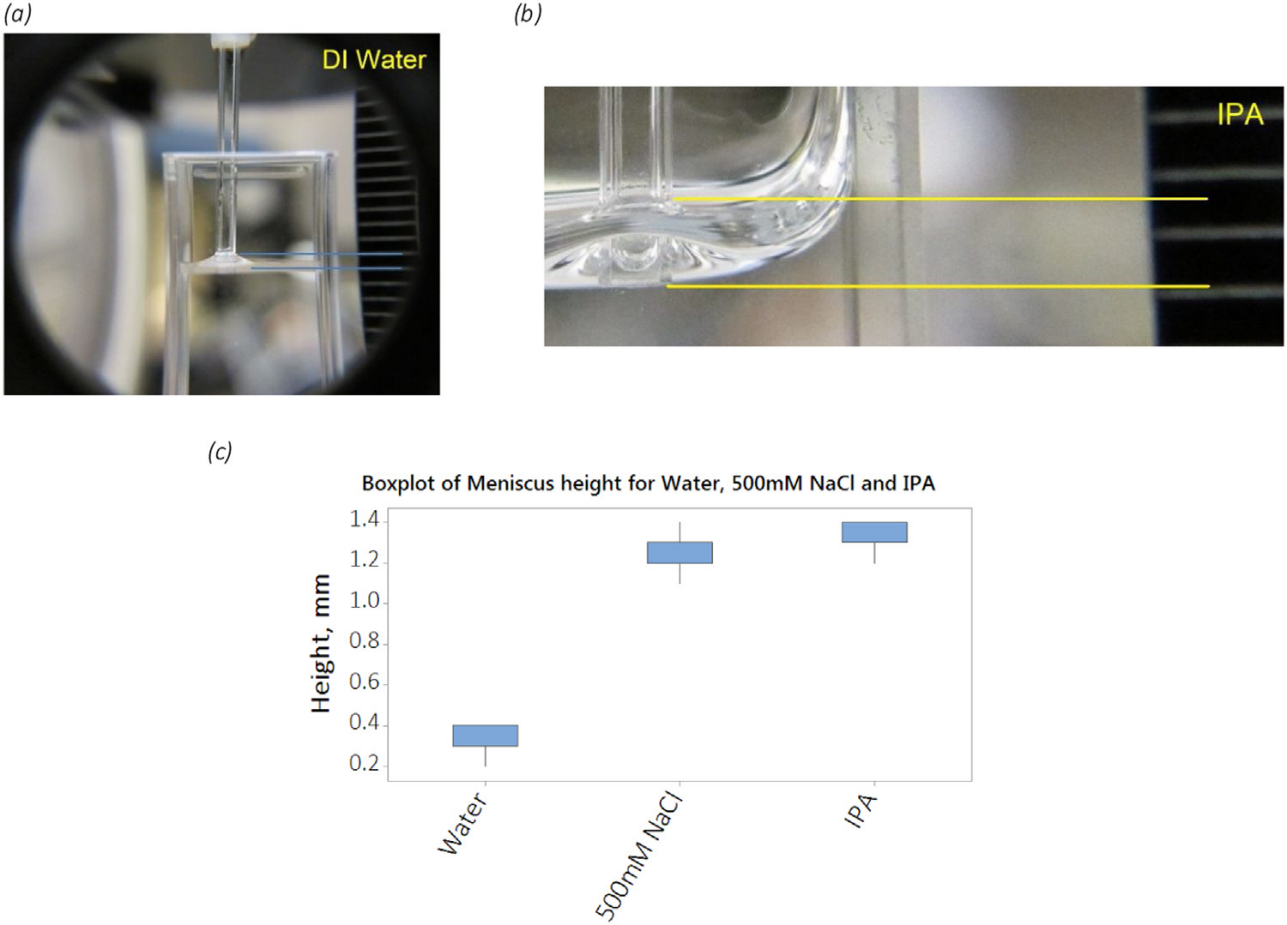
Wicking depth at 20 °C for (a) DI Water, (b) Isopropyl-alcohol(IPA) and (c) estimated from the scale in the images (500 mM not shown) meniscus depth for DI water, 500 mM NaCl and IPA.

Perhaps the most direct way of adding the sample to the capillary is to dip it directly into the bulk sample. However, it is not clear that this is applicable to DLS without validation. The magnitude of the loaded volume will be highly uncertain with factors such as local air temperature and pressure, the temperature and viscosity of the sample and the cleanliness of the internal surfaces of the capillary, etc. The capillary holder presents the sample to the optical beam path at a specific location within the instrument, 4 mm–6 mm above the bottom of the capillary base (and 8.00 mm above the capillary holder base). Also, since dried sample sat on the outside surface of the capillary would act as a static scatterer and therefore reduce the intercept (commonly interpreted as the signal-to-noise in dynamic light scattering measurements) it is desirable to understand how common sample types behave when the capillary is dipped into the bulk, at room temperature. Fig. 1, below, shows the results of such tests at a stable laboratory temperature of 20 °C and at an elevation above sea level of 50 m (Malvern, UK). DI water, filtered to 200 nm is characterized by a meniscus approximately 1 mm in depth, whereas Isopropyl-alcohol and the 500 mM salt solution have increased surface tension on the glass capillary, raising a meniscus of around 1.4 mm. Repeat tests, without magnification, indicate that it is possible to routinely dip the capillary into the dispersant to a maximum of approximately 2 mm, so the effect of the additional meniscus on the ease of filling is considered negligible and filling by dipping and then sealing with a clay slug, is therefore one of the two primary recommended capillary filling strategies, for DLS. In particular, the 1.0 mm by 1.0 mm capillary has a sample volume of 1 μl per filled millimeter along the capillary length and typically 15 mm–30 mm is loaded by dipping and can therefore be used in applications where a few tens of microliters can be spared.

### Filling with a micropipette

**Fig. 2:**
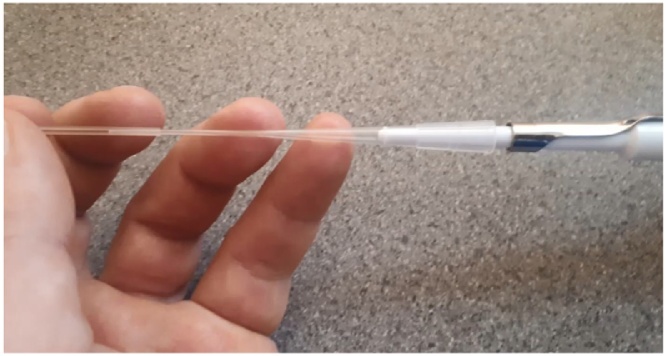
Placing the pipette tip into the capillary.

**Fig. 3:**
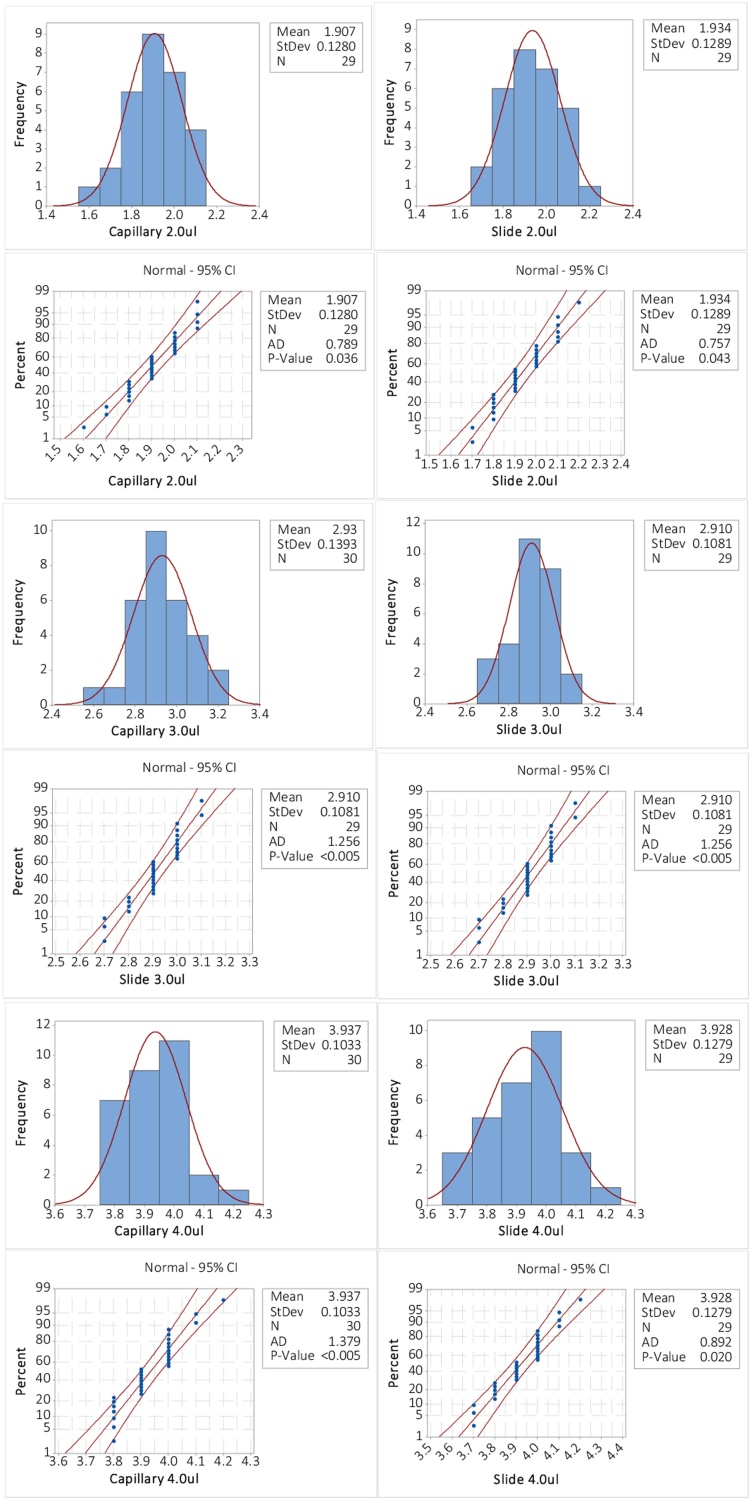
Pipette onto a slide and into the capillary.

Filling with a Geloader tip attached to a micro-pipette – Fig. 2 - implies the use of small volumes of sample, so we have determined the minimum volume that can be reproducibly loaded into the capillary. The data in Fig. 3 are from a comparison study of micro pipetting a specific volume of 200 nm filtered DI water, (18.2 MΩ) onto a microscope slide and into an unused, clean and dry 1.00 mm capillary. The micropipette used was a Gilson Pipetman P200, within its calibration period and the tips were, Geloader™ from Sigma-Aldrich (Z417047).

**Fig. 4:**
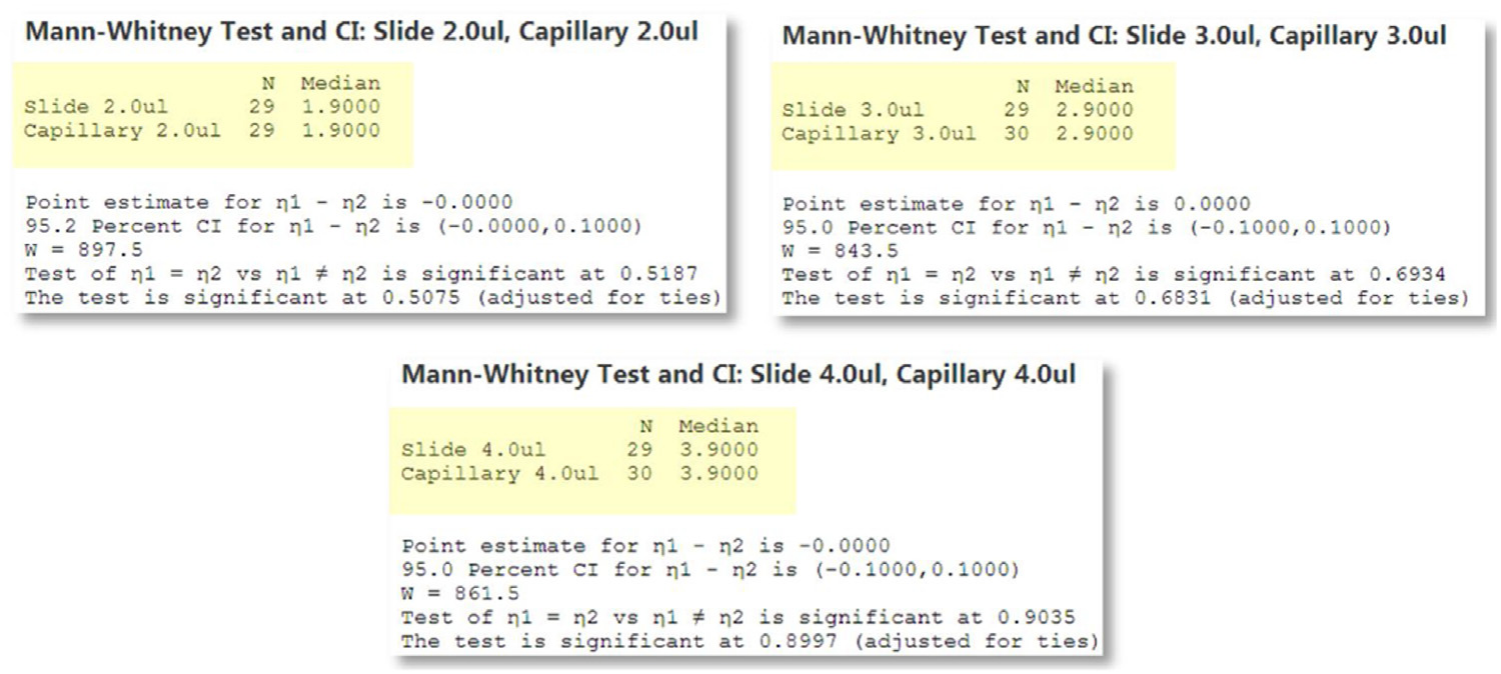
Mann–Whitney tests for differences in Median between slide and capillary: All numerically identical.

The weight of the slide/capillary is measured before and after and the addition of the sample and the volume back-calculated via the density of water at the lab temperature 20 °C, taken [6] as 0.9982 g cm^−3^. Curiously, the test for normality indicates *p*-values all less than 0.05 and therefore the data are all non-normal! A discussion of the hydrodynamic effects within the pipette is far beyond the scope of this work. For now, note that data in the figure are all monomodally distributed and we therefore use the Mann–Whitney test for the median value. The data in Fig. 3 indicate that the medians are identical to 4 decimal places and therefore pipetting these volumes into the capillaries yields identical results to pipetting in a more general sense and we can be confident that valuable samples are transferred from the bulk to the capillary, reproducibly and with minimal loss (Fig. 4).

**Fig. 5:**
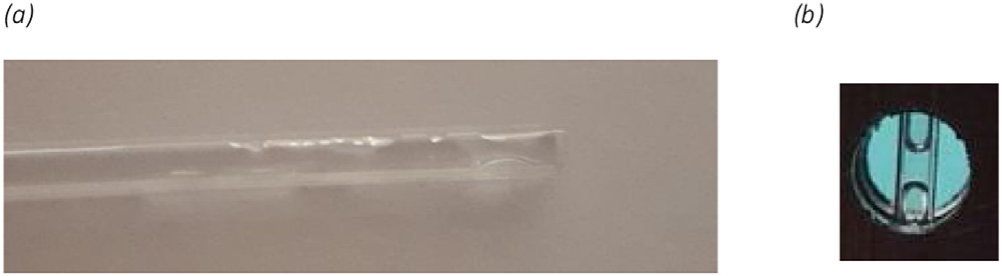
(a) A 2 μl sample that has failed to wick across the capillary cross section, (b) a 3.0 μl sample.

However, a primary issue with pipetting into the 1.0 mm cross section capillary was that significant care was needed to avoid abandoning the drop on the capillary wall(s). Looking to Fig. 5, it was found that volumes of less than 3.0 μl were difficult to wick across the capillary cross section, becoming abandoned on the internal walls. We found that 3.0 μl was a ‘sensible’ minimum specification for the sample volume, although with care smaller volumes were possible.

## Method protocol

### Materials

The DI water was filtered to 200 nm, from a Millipore (US) supply to 18.2 MΩ. The 60 nm polystyrene latex standard was from Thermo-Fisher Scientific (3060A) with a hydrodynamic diameter range of 59 nm–67 nm, dispersed in 150 mM NaCl (S9888-1KG-M, >99.0% Reagent, Sigma-Aldrich, UK) The disposable glass capillaries were from Malvern Instruments (Part No.ZSU2003) with a 1.0 mm × 1.0 mm square internal bore and a wall thickness of 200 μm and the 10.0 mm cuvettes were Sarstedt 67.754, both used throughout this work. A Zetasizer Ultra was used throughout.

### Filling – dipping (non-specific volume)

**Fig. 6:**
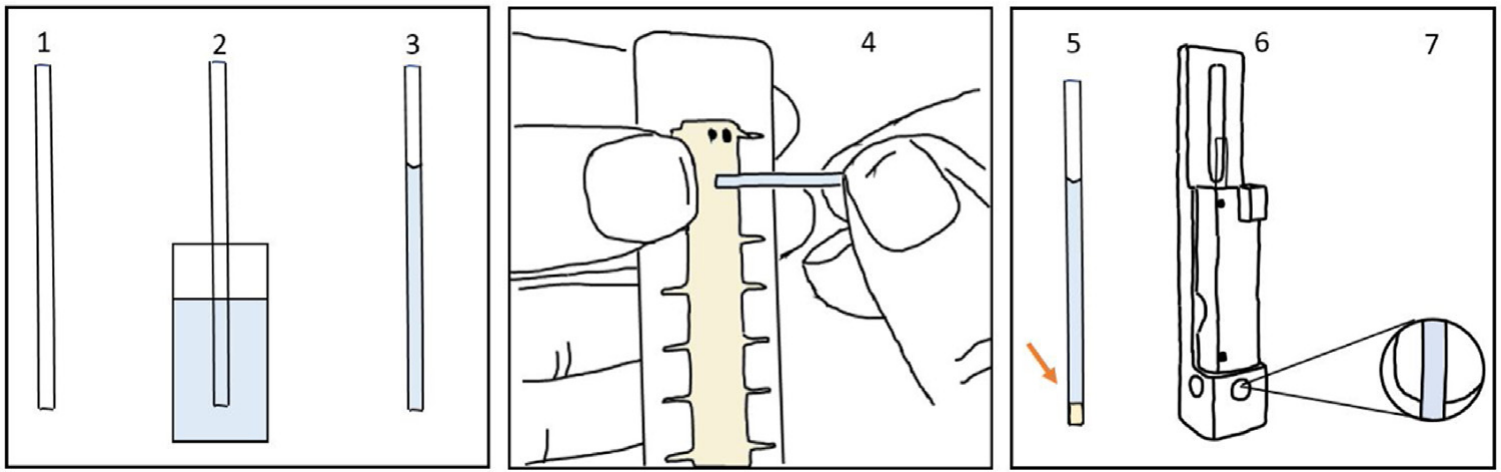
Filling the capillary by air pressure. Place the capillary (1) into the fluid (2) and the capillary fills (3). Add the clay slug to the capillary (4) and place the filled capillary (5) into the capillary holder (6) and check for bubbles in the measurement area (7).

1 Use a clean capillary and keep the filled end of the capillary ends free from finger prints.
2 Dip the capillary into the sample – depending on the air pressure the capillary will be instantly filled with sample between 1.5 cm and 3 cm equivalent to 15–30 μl.
3 Wipe any excess liquid from the bottom end of the capillary.
4 Carefully press the sample end into the sealing putty. Twisting the capillary slightly while pressing it into the putty improves the seal. The depth of the putty is 3 mm.
5 Do not touch the bottom end of the capillary.
6 Place the capillary into the capillary holder with the sealed end at the bottom.
7 Ensure that the capillary is well positioned, and the putty and the meniscus are not visible.
8 Place the holder into the instrument. The Malvern logo should face the front of the instrument (Fig. 6).

### Filling – gel-loading tip (micro-volumes)

**Fig. 7:**
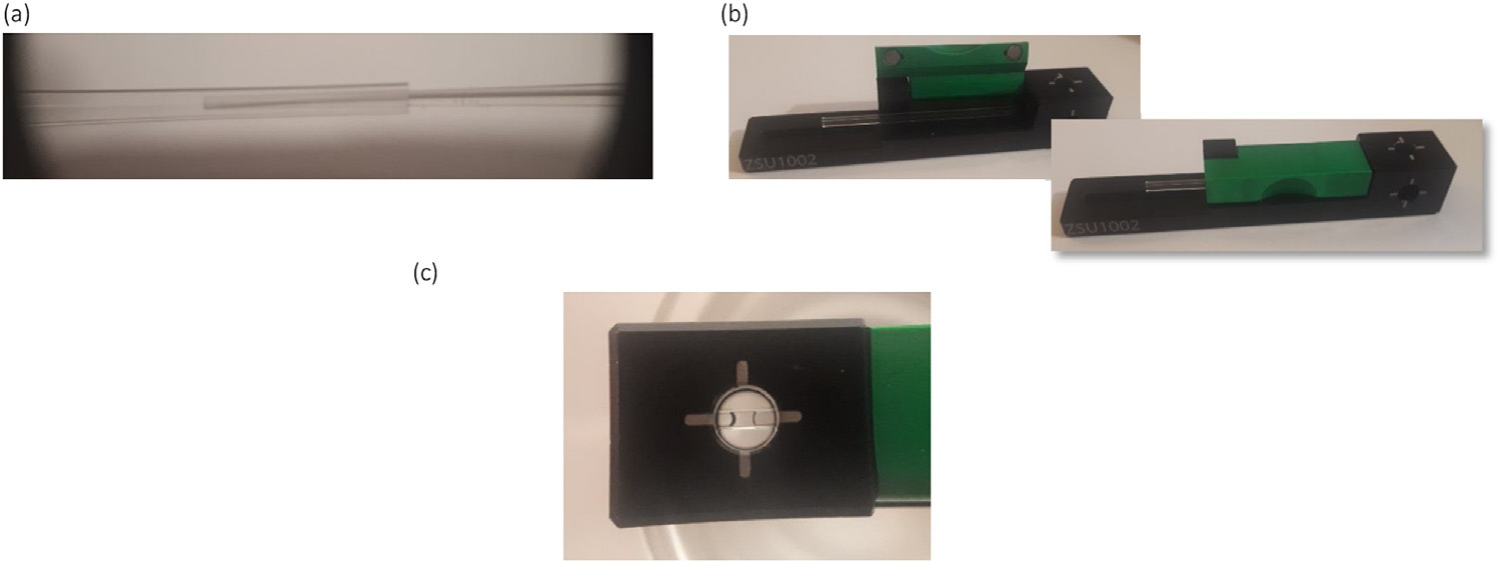
Filling the capillary with a GELoader™ tip, (a) feeding the tip into the capillary, (b) placing the capillary into the ZSU1002 holder, (c) aligning the sample with the cross-hair on the holder.
2 Place the capillary into the holder – Fig. 7(b)
3 Align the slug with the target printed on the outside of the holder – Fig. 7(c) – by sliding the capillary along the holder.
4 Place the holder into the instrument with the back-plate facing forwards to make contact with the mechanical datum and thermal contact surface within the instrument cell area.

1 Fill about 5 mm’s from the end of the capillary – Fig. 7(a). For the smallest volumes, 3 μl to 10 μl, pipette firmly to avoid abandoning the sample slug on the capillary wall.

### Method validation

#### Clay seal

**Fig. 8:**
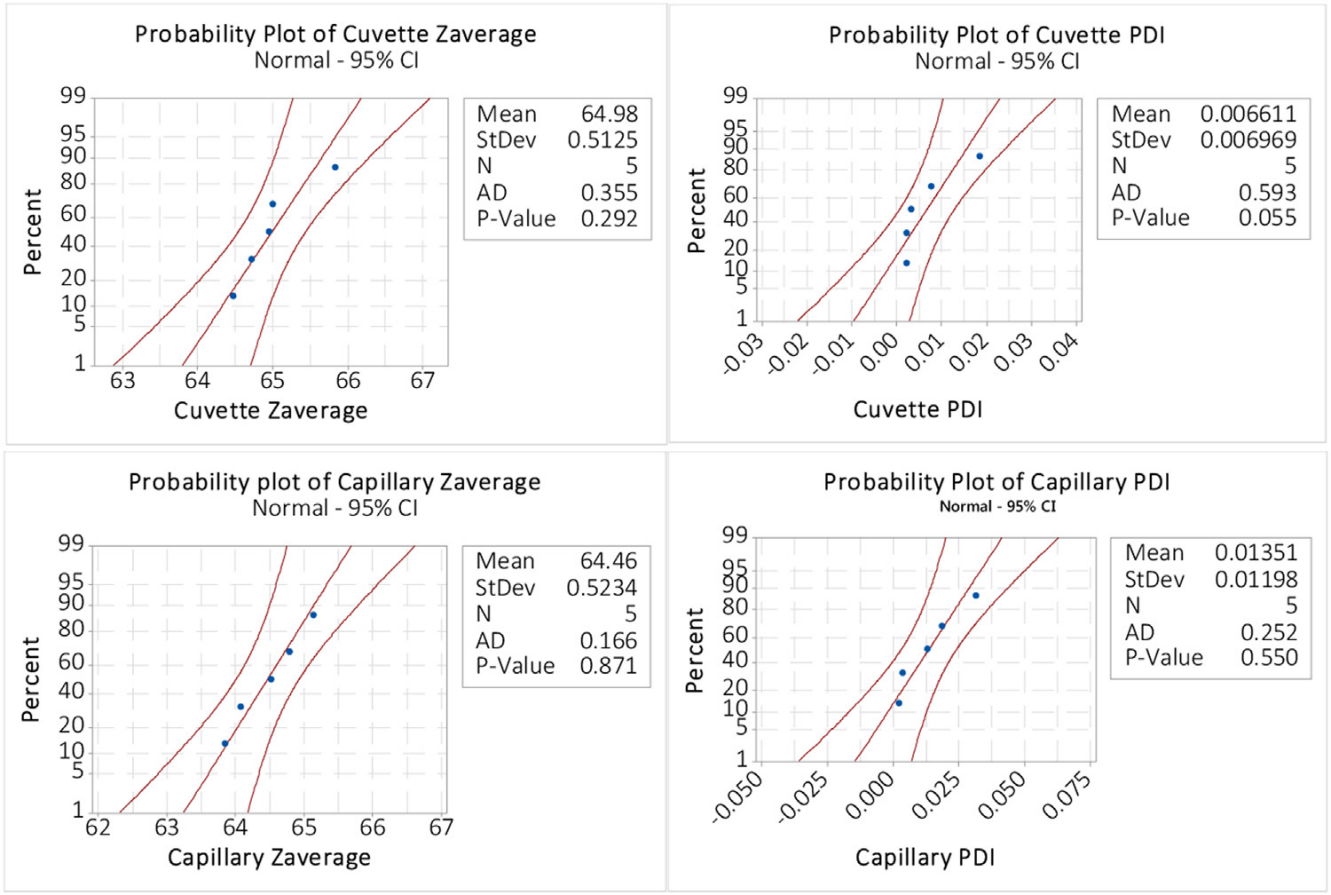
Test for normality for 60 nm latex in 150 mM NaCl.

**Fig. 9:**
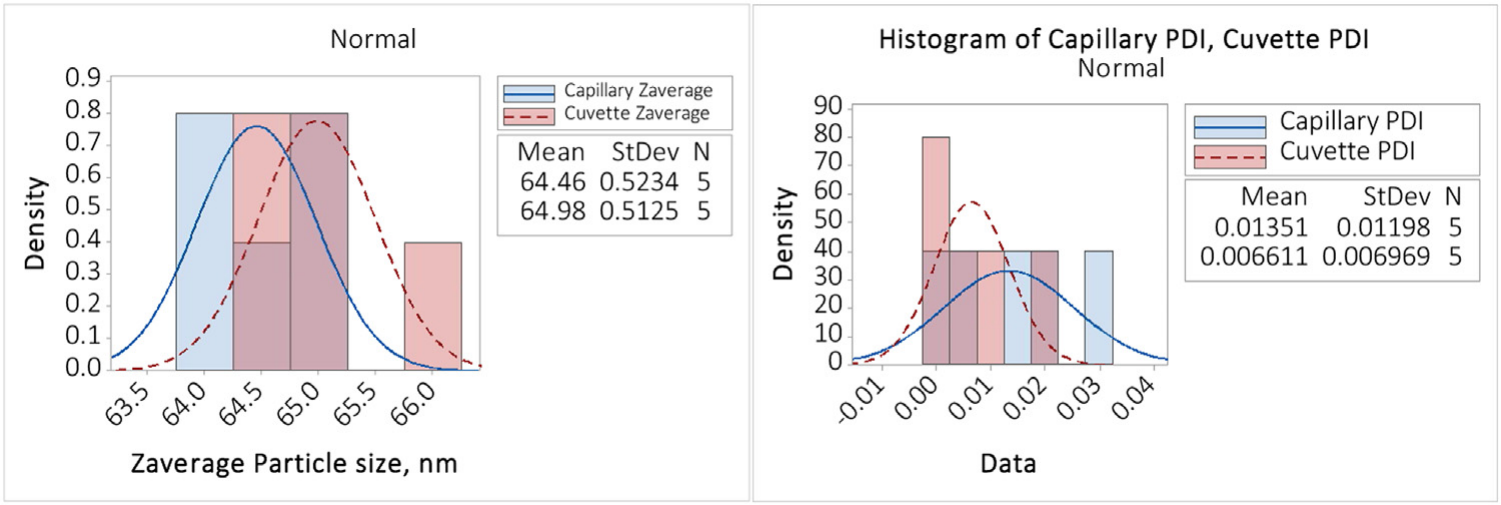
Student *t*-tests – for difference in measurement of dilute 60 nm latex in 150 mM NaCl.

The diluted 60 nm latex sample was characterized using Adaptive correlation, in the ZS Explorer software. Fig. 8 shows that the data are normally distributed with *p*-values for Z_ave_, polydispersity index (PDI) for both 10.0 mm cuvette and 1.0 mm capillary greater than 0.05. Therefore, a student *t*-test comparison of the means is appropriate, as shown in Fig. 9.

Minitab reports back a Zave *p-*value of 0.154 and a PDI *p*-value of 0.308 for the two paired (cuvette and capillary) student *t*-tests. Since both values are >>0.05 (the commonly accepted alpha level) then we fail to reject the null hypothesis for both Z_ave_ and PDI, that the measurements from the cuvette and the clay-sealed capillary are the same. The clay can therefore be considered to have negligible effect on the resulting measurement.

### Pipetted micro-volume

**Fig. 10:**
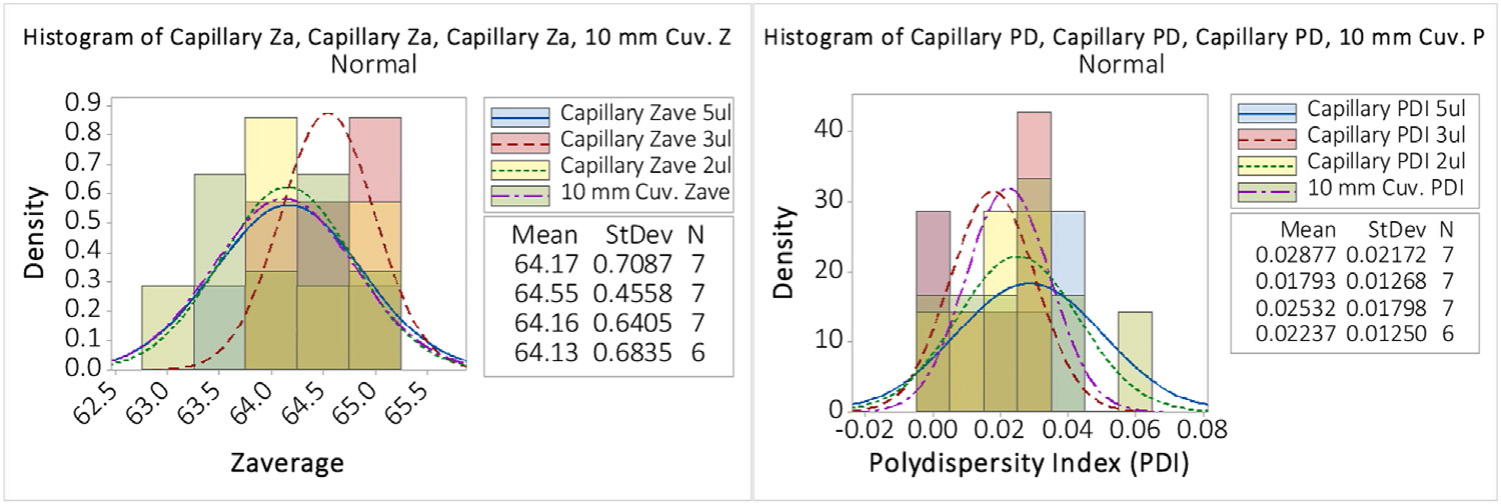
Distribution of (a) Zave and (b) PDI, for 5 μl, 3 μl, 2  μl and a filled 10.0 mm cuvette for the diluted 60 nm latex.

In this section the data from the diluted 60 nm latex are presented from capillary measurements at 5 μl, 3 μl, 2 μl and compared to the results from the 10.0 mm cuvette. The polystyrene latex of hydrodynamic diameter in the range 59 nm–67 nm and an expected PDI of <0.05. The data in Fig. 10 indicate that both the Zave and PDI are in excellent agreement with the 4μl data differing in mean Zave by only 4 Angstroms between cuvette and capillary, well within a single standard deviation. The PDI for the capillary data are fractionally higher than that of the 10.0 mm cuvette, but since all values are <<0.05, universally accepted as a de facto standard for good quality DLS measurements (and significantly lower than the ISO13321: Particle size analysis value of 0.1), then the reported differences are considered negligible. Therefore, the new micro-pipette filling scheme provides a statistically identical measurement to the reference measurements in a 1 cm cuvette, but in a 3 order of magnitude reduction in sample volume.

Finally, we re-iterate that 3 μl is considered to be the minimum reproducible sample volume for the capillary cell. As the data in Fig. 10 indicate, the quality of the result from the 2.0 μl data are equivalent to the 10.0 mm cuvette and the larger capillary volumes, but the uncertainties in filling the capillary with this volume are found to be significantly higher than for the 3 μl case.
